# Effects of the monolaurin based feed additive MGOsyn on methane production, rumen fermentation, and microbial communities using rumen fluid from Hanwoo steers in an *in vitro* study

**DOI:** 10.3389/fmicb.2025.1699688

**Published:** 2025-11-10

**Authors:** Mi Ae Park, Seoyun Son, Da Jung Lim, Hee Seop Yu, Yong Hee Yoon, Seon-Ho Kim, Sang-Suk Lee, Dae-Hyuk Kim, Yangseon Kim

**Affiliations:** 1 Department of Research and Development, Center for Industrialization of Agricultural and Livestock Microorganisms, Jeongeup, Republic of Korea; 2 Jungnongbio, Jeongeup, Republic of Korea; 3 Department of Animal Science and Technology, Sunchon National University, Suncheon, Republic of Korea; 4 Department of Molecular Biology, Department of Bioactive Material Science, Institute for Molecular Biology and Genetics, Jeonbuk National University, Jeonju, Republic of Korea

**Keywords:** MGOsyn, methane reduction, propionate producing bacteria, *in vitro* rumen fermentation, monolaurin

## Abstract

Reducing enteric methane emissions from livestock mitigates environmental impact and improves production efficiency. This initial *in vitro* study investigated the potential of MGOsyn, a new feed additive composed of monolaurin, garlic, and oregano with synergistic antimethanogenic properties, to influence rumen fermentation parameters, methane production, and the structure of bacterial and archaeal communities. The experiment was conducted as a batch culture using rumen fluid collected from Hanwoo steers with ground concentrate as the substrate. MGOsyn, a monolaurin based feed additive (GRAS, Generally Recognized As Safe), was supplemented at three different concentrations. 0% (MGOsyn CON), 0.1% (MGOsyn LOW), and 0.2% (MGOsyn HIGH) of the total fermentation volume. After 24 h of incubation at 39 °C, MGOsyn increased propionate production by 50% in a dose dependent manner, while reducing methane emission by 61%, acetate proportion and the acetate to propionate ratio. Microbial community analyses revealed that MGOsyn effectively altered the ruminal microbiome. The bacterial community exhibited an increased relative abundance of succinate and propionate producing bacteria, such as *Succinivibrio, Succiniclasticum, Candidatus Saccharimonas, Succinivibrionaceae* UCG-002, and *Prevotella* 7 which are involved in hydrogen sink pathways following MGOsyn supplementation. Considering the archaeal community, the abundance of *Methanomethylophilaceae* decreased with MGOsyn supplementation compared with that in the control, whereas *Methanosphaera* increased in the high MGOsyn supplementation group. To further explore the underlying mechanisms of methane mitigation, we performed correlation and intercorrelation analyses between fermentation parameters and microbial taxa, along with functional predictions of relevant metabolic pathways. Succinate/propionate producing bacteria showed strong positive correlations with MGOsyn supplementation and propionic acid production, and strong negative correlations with acetic acid production, the acetate to propionate ratio, and methane production. These findings suggest that MGOsyn effectively mitigates methane emissions by stimulating propionate formation through the hydrogen sink pathway. By altering the ruminal microbial community toward enhanced propionate production, MGOsyn shows promise as a functional feed additive for improving rumen fermentation efficiency and reducing methane output in ruminants.

## Introduction

Ruminants are major sources of greenhouse gas emissions within the livestock sector, largely due to enteric fermentation, which is responsible for roughly 80% of the sector's emissions. Overall, emissions from ruminants contribute an estimated 16% to total anthropogenic greenhouse gases worldwide (Tseten et al., 2022). Enteric methane (CH_4_) is primarily produced in the rumen by methanogenic archaea that convert hydrogen (H_2_) and carbon dioxide (CO_2_) into CH_4_. These methanogens interact with other ruminal microbes, including protozoa, bacteria, and fungi, through interspecies H_2_ transfer, where methanogens utilize H_2_ produced by other microbes (Patra et al., 2017; Patra and Saxena, 2010). In contrast, microbiome analyses have indicated that numerous bacteria are associated with variations in CH_4_ production in ruminants, and differences in bacterial diversity and composition among breeds or dietary regimens can influence methanogenesis by altering fermentation pathways and hydrogen availability (Tapio et al., 2017). This enteric CH_4_ emission is also associated with a dietary energy loss of up to 12%, thereby reducing feed efficiency (Cuervo et al., 2025). Due to the detrimental impacts of methane (CH_4_) on both the environment and livestock productivity, considerable efforts have been directed toward its reduction in recent decades. Various approaches have been explored to lower CH_4_ emissions from ruminants, including strategies that target rumen fermentation, microbial community composition, and overall rumen function. Among these, dietary interventions are particularly influential, as they can directly modify fermentation pathways and the resulting metabolic end products. Notably, one review emphasized that shifting rumen fermentation profiles remains one of the most effective methods for mitigating CH_4_ production (Haque, 2018).

Following the prohibition of antibiotics and synthetic compounds in livestock diets by the EU ban in 2006, researchers in the fields of ruminant nutrition and animal science have turned their attention toward natural feed additives as sustainable and eco-friendly alternatives. Among these, plant derived compounds, such as tannins, saponins, and essential oils, have garnered considerable attention owing to their potential to modulate ruminal microbial populations and fermentation patterns. These compounds can enhance beneficial microbial activity and reduce enteric CH_4_ production by altering the composition and functionality of rumen microbial communities (Martinez-Fernandez et al., 2013; Patra et al., 2011; Patra and Saxena, 2010; Pawar et al., 2014). Researchers continue to explore new compound mixtures that can effectively suppress CH_4_ production without negatively affecting the production of volatile fatty acids (VFAs; Bature et al., 2024).

Numerous studies have demonstrated the potential of natural plant-derived compounds to mitigate methane emissions from ruminants. Lauric acid has been reported to reduce CH_4_ production by 20–90% *in vitro* and 10–20% *in vivo*, garlic by up to 75% *in vitro* and 10–20% *in vivo*, and oregano by 10–90% *in vitro*, depending on experimental conditions and factors such as diet, dosage, duration, and animal species (Kim et al., 2018; Ma et al., 2016; Malyugina et al., 2025; Panthee et al., 2017; Soliva et al., 2003; Tseten et al., 2022). These compounds are thought to act primarily through their antimicrobial properties, which alter the composition and activity of rumen microbial communities, thereby suppressing methanogenesis.

Among these, monolaurin has received limited research attention with only a few studies to date examining its effects on rumen fermentation and methane production (Klevenhusen et al., 2009, 2011). Furthermore, its specific mechanisms of action, particularly in relation to the rumen microbiome and metabolic pathways involved in methane synthesis, remain poorly understood and its standalone efficacy and potential synergistic effects with other phytogenic compounds are still underexplored. In response to this research gap, our team developed a novel, eco-friendly natural feed additive designated as MGOsyn composed of monolaurin, a monoglyceride derived from lauric acid and naturally found in coconut oil and human breast milk, together with garlic (*Allium sativum*) powder and oregano (*Origanum vulgare*) powder. Each component is known for its bioactive properties, including antimicrobial activity, modulation of fermentation, and inhibitory effects on methane (CH_4_) production (Ankri and Mirelman, 1999; Barker et al., 2019; Busquet et al., 2005; Ma et al., 2016; Nitbani et al., 2022; Panthee et al., 2017; Tseten et al., 2022; Vennard et al., 2024). To evaluate the effectiveness of MGOsyn prior to *in vivo* application, we conducted an *in vitro* rumen fermentation study using rumen fluid collected from Hanwoo cattle, incubated at 39 °C for 24 h. This experimental setup allowed us to assess the impact of MGOsyn on methane production, key rumen fermentation parameters, and the composition of the rumen microbial community. Furthermore, to investigate the potential mechanisms underlying its effects, we conducted correlation and intercorrelation analyses between rumen fermentation parameters, including metabolite profiles and microbial taxa, and performed functional predictions of relevant metabolic pathways based on microbial profiles inferred using PICRUSt2 (Langille et al., 2013) and MaAsLin3 (Nickols et al., 2024). These integrative analyses enabled us to gain valuable insights into how MGOsyn may modulate microbial metabolic activities, particularly those associated with hydrogen utilization and propionate production pathways, ultimately contributing to methane mitigation.

Through this study, we aim to provide foundational evidence for monolaurin based feed additive MGOsyn's efficacy and insights into rumen modulation by natural compounds.

## Materials and methods

### Rumen fluid collection

Rumen fluid was obtained from two Hanwoo steers (Korean native breed; average body weight: 600 ± 50 kg) in the early morning prior to feeding, using a stomach tube connected to a vacuum pump. The collected rumen fluid was transferred into an insulated container and immediately transported to the laboratory. Upon arrival at the laboratory, the rumen fluid was filtered through four layers of cheesecloth and maintained in a 39 °C water bath. It was continuously flushed with CO_2_ for 20 min prior to being used in the *in vitro* rumen fermentation assays. All procedures related to animal handling and sample collection were reviewed and approved by the Institutional Animal Care and Use Committee of Sunchon National University (approval ID: SCNU-IACUC-2022-06) (Berdos et al., 2023). The study followed the guidelines outlined in the ARRIVE (Animal Research: Reporting of *In Vivo* Experiments, https://arriveguidelines.org/), with all possible measures taken to minimize animal distress.

### Experimental design and *in vitro* rumen fermentation

Three experimental groups were prepared based on the MGOsyn supplementation dose: 0% (MGOsyn CON), 0.1% (MGOsyn LOW), and 0.2% (MGOsyn HIGH) of the total fermentation volume. Each experimental group contained 1 g of ground concentrate passed through a 1 mm sieve as the substrate, which was placed in a 250 mL ANKOM fermentation bottle (ANKOM Technology, USA). The chemical composition of the substrate (concentrate feed) is provided in Supplementary Table S1. The experiment was conducted in three independent runs, with three replicate samples per treatment group in each run. MGOsyn was directly supplemented into the bottles at either 0.1% or 0.2% of the total fermentation volume, depending on the treatment. MGOsyn comprised a mixture containing eight parts monolaurin (98% purity; TCI, Japan), one part garlic (*Allium sativum*) powder (100% purity, commercial product), and one part oregano (*Origanum vulgare*) powder (100% purity, commercial product), selected based on considerations of methane reduction (Supplementary Figure S1), palatability, and cost-effectiveness. A total of 100 mL of rumen fluid and McDougall's buffer mixture (1:2 ratio) was added to each bottle under continuous CO_2_ flushing. The bottles were subsequently incubated at 39 °C for 24 h (Supplementary Figure S2). At the end of incubation, pH was measured using a pH meter (SevenCompact pH/Ion meter, METTLER TOLEDO, USA), and the culture fluid was aliquoted into 1.5 mL microtubes and stored at −20 °C for further analysis.

### Analytical conditions for methane gas analysis using GC-FID/methanizer

The methane gas concentrations were quantitatively determined using a gas chromatography (GC) system (Nexis GC-2030, Shimadzu, Japan). The system was equipped with a flame ionization detector (FID) coupled with a methanizer. Chromatographic separation was conducted using an MXT Guard column (0.5 mm ID) connected in series with an HP-PLOT/Q column (30 m x 0.55 mm, 40 μm). The oven temperature of the column was initially set at 40 °C and maintained for 10 min. Samples were injected in split mode with a split ratio of 5, and the injector temperature was maintained at 100 °C. For detection, the methanizer was operated at 350 °C. The FID temperature was set at 100 °C, with N_2_, H_2_, and air make up gases supplied at 24, 28, and 200 mL/min, respectively.

### Analysis of VFAs

For the quantification of eight VFAs, including acetic, propionic, butyric, isobutyric, lactic, valeric, isovaleric, and 2-methylbutyric acids, we employed a derivatization method using 3-nitrophenylhydrazine (3-NPH), followed by ultra performance liquid chromatography tandem mass spectrometry (UPLC-MS/MS) analysis (Han et al., 2015). The UPLC-MS/MS system used was an ACQUITY UPLC I-Class/Xevo TQ-S micro (Waters, USA) equipped with an electrospray ionization (ESI) source and operated in negative ion mode. Chromatographic separation was achieved on an ACQUITY UPLC BEH C18 column (2.1 × 100 mm, 1.7 μm, Waters, USA). The mobile phase comprised water (A) and acetonitrile (B), both containing 0.1% formic acid, delivered at a flow rate of 0.5 mL/min using a gradient elution program. Quantification was conducted in the multiple reaction monitoring (MRM) mode, and the specific MRM transitions (Q1/Q3, m/z) for each VFAs were as follows: acetic acid (194.00/137.00), propionic acid (208.00/137.00), butyric acid (220.00/137.00), isobutyric acid (222.00/137.00), lactic acid (224.13/137.00), valeric acid (236.00/137.00), isovaleric acid (236.00/137.00), and 2-methylbutyric acid (236.00/137.00).

### DNA extraction

DNA was isolated from *in vitro* rumen fermentation samples using the ARA MagNA Stool DNA Isolation Kit (LAS, Korea). 200 μL aliquot of each sample was placed into 2 mL centrifuge tube, followed by the addition of 20 μL of proteinase K (40 mg/mL) and 300 μL of FL1 lysis buffer. The mixture was subjected to vortexing for 10 min, then centrifuged at 12,000 × g for 2 min to eliminate debris. From the resulting supernatant, 400 μL was transferred and combined with 400 μL of FB2 binding buffer and 20 μL of magnetic beads. DNA purification was carried out in accordance with the kit manufacturer's protocol (LAS, Korea). The final DNA extracts were preserved at −20 °C until further analysis.

### Library preparation and sequencing

For microbial community analysis, a 16S rRNA gene sequencing library was prepared by amplifying the V3–V4 hypervariable regions. The amplification was performed using KAPA HiFi HotStart ReadyMix (KAPA Biosystems, USA). Resulting amplicons were purified with ARAClean magnetic beads (LAS, Korea). The first round of PCR used region specific primers compatible with MGI adapters and indices (Forward: 5′-GGCTCACAGAACGACATGGCTACGATCCGACTTCCTACGGGNGGCWGCAG-3′; Reverse: 5′-TTGTCTTCCTAAGACCGCTTGGCCTCCGACTTGACTACHVGGGTATCTAATCC-3′). After bead based purification, a second PCR was performed using indexing primers from the MGIEasy UDB Primers Adapter Kit A (MGI, Shenzhen, China), applying a limited number of cycles to minimize amplification bias. The final PCR products were verified by agarose gel electrophoresis and quantified using the Qubit dsDNA High Sensitivity Assay Kit (Invitrogen, USA) with a Qubit 4.0 fluorometer. For library circularization, the pooled amplicons were treated with the MGIEasy Dual Barcode Circularization Module (MGI, China) at 37 °C for 30 min. The circularized DNA underwent a digestion step at the same temperature and duration, followed by a purification process. To form DNA nanoballs (DNBs), the circularized library was incubated at 30 °C for 15 min with a DNB enzyme mix. The final library concentration was assessed using the Qubit ssDNA HS Assay Kit (Invitrogen, USA). Sequencing was performed on the MGIseq platform (MGI, China) using a paired end 300 bp read configuration.

### Sequencing bioinformatics and microbiome analysis

The QIIME2 (Bolyen et al., 2019) DADA2 package (version 2019.4.0; Callahan et al., 2015) was used to denoise the paired end sequences, duplicate them, merge forward and reverse reads, and filter chimeras. Representative sequences were classified using QIIME2 scikit-learn (version 2019.4.0; Pedregosa et al., 2011) with a pretrained classifier trained on the SILVA (database release 132; Quast et al., 2013), and multiple sequence alignments were conducted using QIIME2 MAFFT (version 2019.4.0; Katoh and Standley, 2013). Downstream data analysis was conducted in the R statistical environment (R Core Team, 2023) using a combination of custom scripting with the microbiome, phyloseq, vegan, ggplot2, ALDEx2, MaAsLin 3, and Microeco packages.

Relative abundance was analyzed using the phyloseq (version 1.46.0; McMurdie and Holmes, 2013) and microbiome (version 1.24.0) packages in R (Lahti and Shetty, 2017). α-diversity (Shannon, 1948; Simpson, 1949) was assessed using the phyloseq package in R. β-diversity (Whittaker, 1960), which measures the dissimilarity of the microbial community composition between samples, was characterized using the Bray–Curtis index. A principal coordinate analysis (PCoA) plot was used to visualize the Bray–Curtis dissimilarity among the samples.

Spearman's correlations analysis was conducted at the genus level to explore associations among group specific microbial communities. Spearman correlation (*P* < 0.05) was conducted in R, and the correlation network was visualized using Gephi software (Bastian et al., 2009).

We also predicted the functional profiles of the microbial communities from our 16S rRNA gene data using the phylogenetic investigation of communities by reconstruction of unobserved states (PICRUSt; Langille et al., 2013). The Microeco package (version 1.7.1; Liu et al., 2021) in R was used for the analysis and visualization of the relative abundance and ALDEx2 analysis (Fernandes et al., 2013). The MaAsLin3 package (Nickols et al., 2024) in R was used for the analysis and visualization of PICRUSt functional pathway.

### Statistical analysis

R statistical software was used for further statistical analyses, primarily using the vegan package. Statistical analysis of α-diversity indices among groups was conducted using the Kruskal-Wallis test (Kruskal and Wallis, 1952). Pairwise comparisons between groups were performed using Wilcoxon rank-sum tests (Wilcoxon, 1945), and resulting *p*-values were adjusted for multiple comparisons using the Holm–Bonferroni method (Holm, 1979). Differences in β-diversity were assessed using permutational multivariate analysis of variance (PERMANOVA) with 999 permutations based on Bray–Curtis dissimilarities, implemented with the Adonis2 function in the vegan package of R (version 2.6-4; Oksanen et al., 2018). Since only overall group comparisons were conducted, *p*-values were not adjusted for multiple testing. Normality was assessed using the Shapiro-Wilk test. For microbial taxa that did not meet the assumption of normality, significant differences among groups were assessed using the non-parametric Kruskal–Wallis test, followed by pairwise comparisons with the Wilcoxon rank-sum test or Dunn's multiple comparison test (Dunn, 1964). For other data that were normally distributed, statistical differences among multiple groups were analyzed using one way analysis of variance (ANOVA), with Tukey's honestly significant difference (HSD) test applied as a *post-hoc* analysis.

## Results

### Impact of MGOsyn on fermentation parameters in the rumen

**Figure 1:**
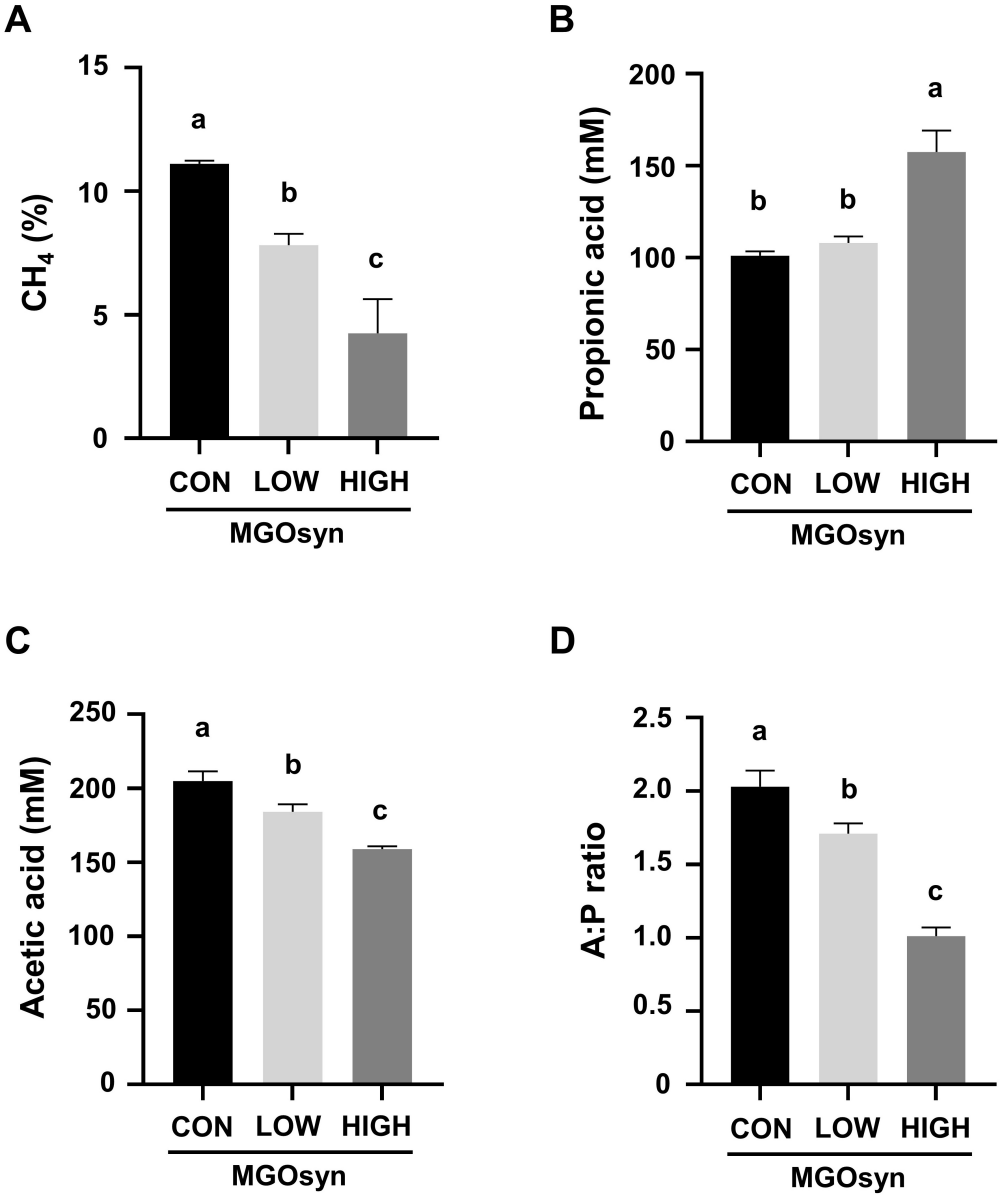
Effects of MGOsyn supplementation on rumen fermentation characteristics. **(A)** Percentage of methane (CH_4_) in the total gas produced after 24 h of *in vitro* fermentation with MGOsyn supplementation. **(B)** Concentration of propionic acid (mM), a key volatile fatty acid associated with energy efficiency and hydrogen utilization. **(C)** Concentration of acetic acid (mM), the most abundant volatile fatty acid produced during rumen fermentation. **(D)** Ratio of acetic acid to propionic acid (A:P ratio), an important indicator of rumen fermentation pattern shifts. Bars with different superscript letters indicate statistically significant differences among treatment groups (*P* < 0.05).

Significant alterations in rumen fermentation profiles were observed following MGOsyn treatment, as presented in Table 1. The percentage of CH_4_ in the produced gas significantly decreased with increasing doses of MGOsyn (P = 0.0002), showing reductions up to 30% in the MGOsyn LOW group and 61% in the MGOsyn HIGH group compared to the control group (*P* < 0.05; Figure 1A, Table 1). Enhanced supplementation of MGOsyn led to elevated propionic acid levels with the MGOsyn HIGH group showing a marked and statistically significant increase compared to the control and MGOsyn LOW groups (*P* = 0.0001; Figure 1B, Table 1). However, the acetic acid concentration decreased among the groups *(P* < 0.0001) with increasing MGOsyn doses (Figure 1C, Table 1). MGOsyn did not affect the concentrations of lactic acid or total VFAs among the groups. The ratio of acetate to propionate (A:P) declined significantly in response to increasing levels of MGOsyn supplementation (*P* < 0.0001; Figure 1D, Table 1).

**Table 1:** Rumen fermentation characteristics after 24 h of *in vitro* incubation.

**Parameter**	**Experimental groups**			***P*-value**
	**CON**	**MGOsyn LOW**	**MGOsyn HIGH**	
pH	6.25 ± 0.01^b^	6.29 ± 0.01^a^	6.17 ± 0.01^c^	< 0.0001
Total gas production (mL)	157.20 ± 2.90^a^	147.29 ± 4.10^a^	103.53 ± 4.78^b^	< 0.0001
CH_4_/DM (mL/g)	17.45 ± 0.34^a^	11.53 ± 1.04^b^	4.45 ± 1.63^c^	< 0.0001
CH_4_/Total gas production (mL/mL)	0.111 ± 0.001^a^	0.078 ± 0.005^b^	0.043 ± 0.014^c^	0.0002
CH_4_ (%)	11.11 ± 0.13^a^	7.82 ± 0.46^b^	4.25 ± 1.38^c^	0.0002
Acetic acid (mM)	204.87 ± 6.42^a^	184.08 ± 5.0^b^	158.93 ± 1.95^c^	< 0.0001
Propionic acid (mM)	101.09 ± 2.28^b^	107.92 ± 3.57^b^	157.48 ± 11.55^a^	0.0001
Butyric acid (mM)	67.48 ± 1.25^ab^	76.99 ± 2.00^a^	56.34 ± 9.19^b^	0.0105
Isobutyric acid (mM)	3.12 ± 0.01^a^	2.84 ± 0.13^b^	2.67 ± 0.08^b^	0.0023
2-Methylbutyric acid (mM)	6.14 ± 0.29^b^	6.88 ± 0.22^a^	4.89 ± 0.32^c^	0.0004
Lactic acid (mM)	0.30 ± 0.08^ns^	0.25 ± 0.05^ns^	0.28 ± 0.02^ns^	0.5725^ns^
Valeric acid (mM)	10.70 ± 0.33^ab^	10.78 ± 0.60^a^	9.37 ± 0.66^b^	0.0342
Isovaleric acid (mM)	6.37 ± 0.54^ns^	6.14 ± 0.06^ns^	5.66 ± 0.22^ns^	0.1012^ns^
Total VFAs (mM)	400.08 ± 11.19^ns^	395.88 ± 11.66^ns^	395.62 ± 24.00^ns^	0.9355^ns^
A:P ratio	2.03 ± 0.11^a^	1.71 ± 0.07^b^	1.01 ± 0.06^c^	< 0.0001

^a, b, c^Values within the same row with different superscripts are significantly different (*P* < 0.05). DM, dry matter; VFAs, volatile fatty acids; A:P, acetate to propionate ratio.

### Impact of MGOsyn supplementation on bacterial diversity and community structure

**Figure 2:**
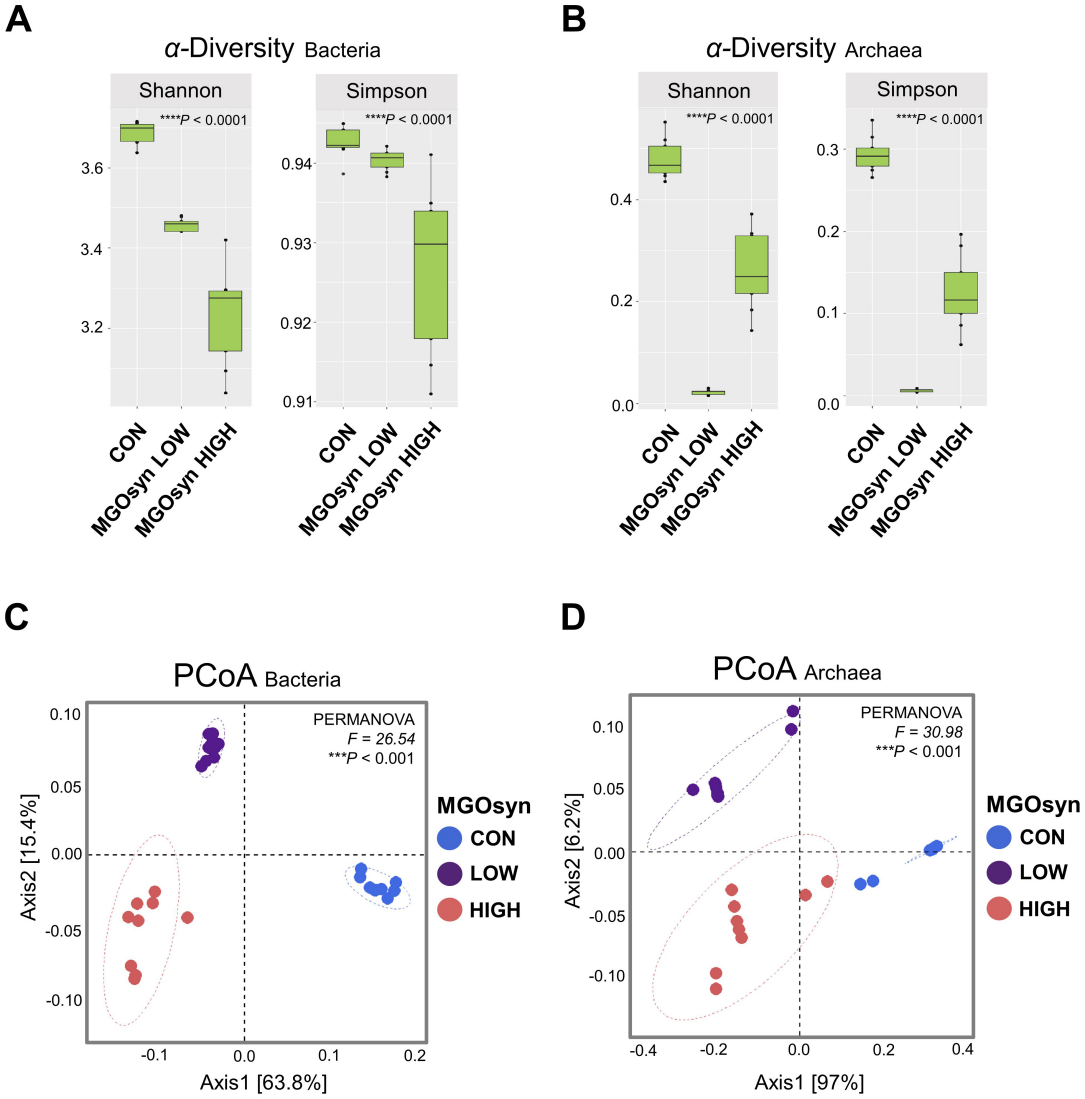
α**-**diversity and β-diversity of microbial community composition in response to MGOsyn supplementation. **(A)** α-diversity indices (Shannon and Simpson) of the bacterial community under different levels of MGOsyn supplementation. **(B)** α-diversity indices of the archaeal community under different levels of MGOsyn supplementation. **(C)** Plot of principal coordinate analysis (PCoA) based on Bray**–**Curtis dissimilarity illustrating differences in the bacterial community composition at the genus level across the treatment groups. Each point represents an individual sample, and the distance between points reflects the differences in microbial composition. The MGOsyn supplemented groups (LOW and HIGH) clustered separately from the control group, indicating distinct bacterial community structures. **(D)** PCoA plot based on Bray**–**Curtis dissimilarity showing the archaeal community composition at the genus level. Statistical significance: ****P < 0.0001, ***P < 0.001.

**Figure 3:**
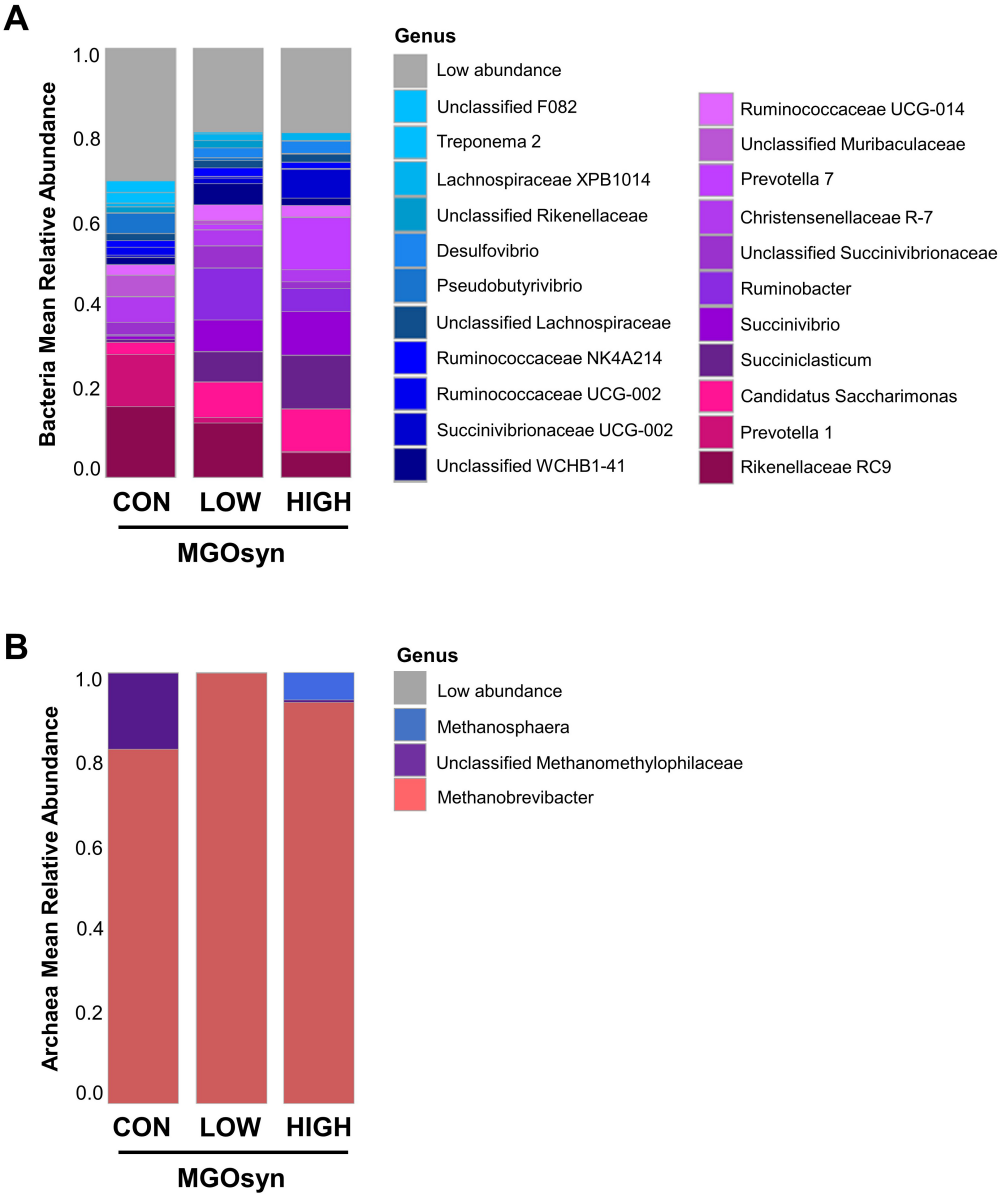
Mean relative abundances of microbial taxa at the genus level. **(A)** Bacterial community composition at the genus level across treatment groups. The bar plots represent the average relative abundances of the dominant bacterial genera detected in each group, indicating shifts in microbial profiles associated with MGOsyn supplementation. Genera with relative abundances below a certain threshold (< 1%) were grouped as Low abundance for clarity. **(B)** Archaeal community composition at the genus level. The bar plots illustrate the average relative abundances of archaeal genera across treatments, indicating changes in methanogenic archaeal populations in response to MGOsyn supplementation.

**Figure 4:**
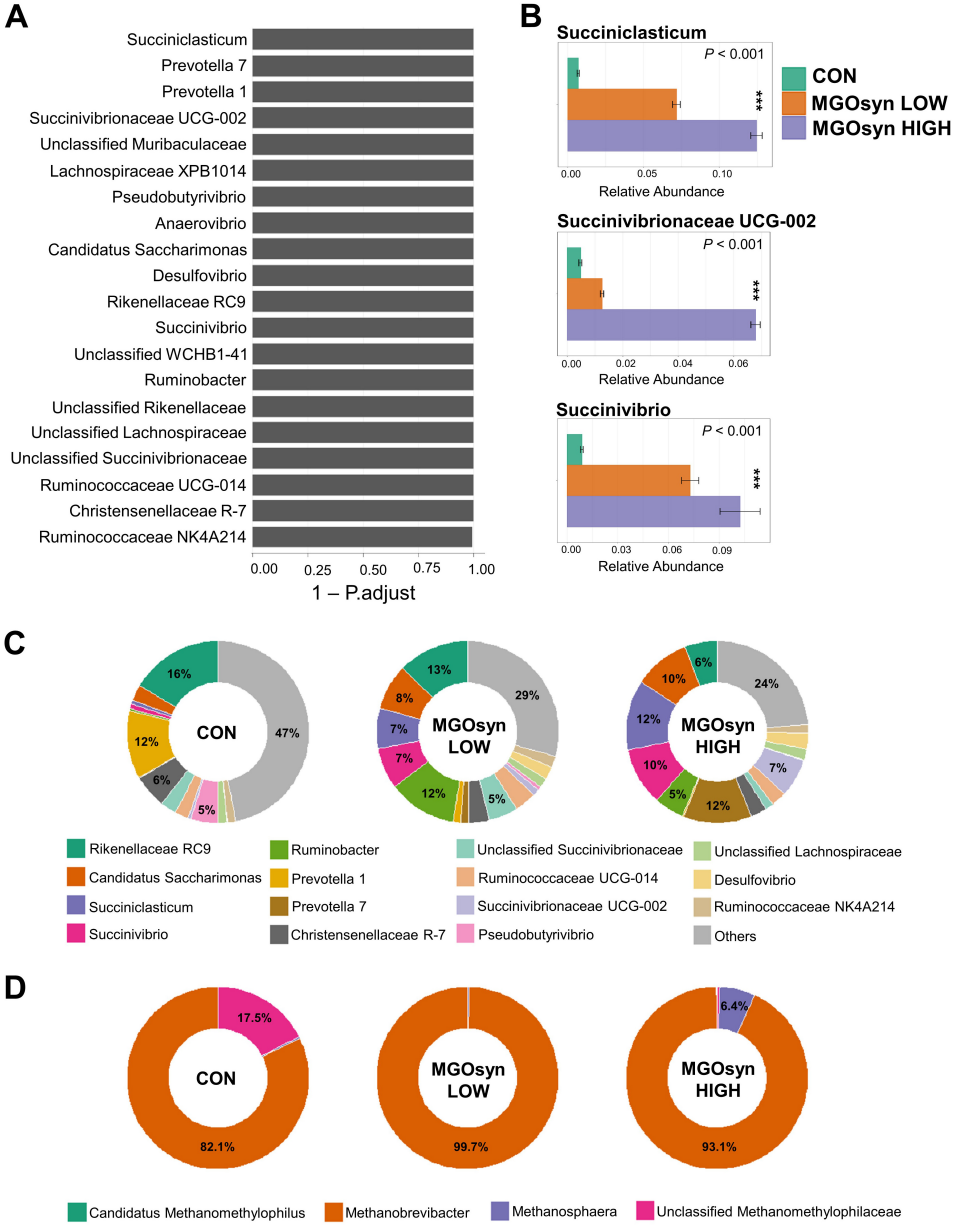
Effects of MGOsyn supplementation on bacterial and archaeal community composition. **(A)** Differentially abundant taxa among MGOsyn treatment groups identified using ALDEx2 analysis, based on centered log-ratio (CLR) transformed abundance data. Taxa with significant differences (adjusted *P* < 0.05) are shown, indicating key genera that responded to MGOsyn supplementation. **(B)** Relative abundances of representative taxa, including *Succiniclasticum, Succinivibrionaceae* UCG-002, and *Succinivibrio*. Statistical analysis was performed using the Kruskal–Wallis rank-sum test. ***P < 0.001. **(C)** Relative abundance of the top 15 bacterial genera presented as donut charts. The charts illustrate shifts in the bacterial community composition across the treatment groups (CON, MGOsyn LOW, and MGOsyn HIGH). **(D)** Relative abundance of the top four archaeal genera shown in donut charts. These charts depict changes in the methanogenic archaeal populations.

The α-diversity indices, including the Shannon (richness and evenness) and Simpson (dominance and evenness) indices of the bacterial community, were significantly influenced by MGOsyn supplementation (*P* < 0.0001; Figure 2A, Table 2). Furthermore, β-diversity analysis at the genus level revealed that the control samples clustered separately from the MGOsyn supplemented groups (*F* = 26.539, *P* < 0.001; Figure 2C). MGOsyn supplementation resulted in a notable increase in *Succinivibrio* at the genus level indicating an overall alteration in the microbial community structure. This increase was significant compared with that in the control (< 1%), MGOsyn LOW (7%), and MGOsyn HIGH (10%) groups (*P* < 0.0001). Similarly, the relative abundance of *Succiniclasticum* increased in the MGOsyn LOW (7%) and MGOsyn HIGH (12%) groups compared with that in the control (< 1%; *P* < 0.0001). The relative abundance of *Candidatus Saccharimonas* increased in the MGOsyn LOW (8%) and MGOsyn HIGH (10%) groups compared with that in the control (3%; *P* < 0.0001). *Rikenellaceae* RC9 and *Prevotella* 1 were significantly decreased in the MGOsyn supplemented groups compared with the control, whereas *Prevotella* 7 was increased in the MGOsyn HIGH (12%) group relative to the control (< 1%; *P* < 0.0001; Figures 3, 4, Table 3).

**Table 2:** α-Diversity of the microbial community.

**Parameter**	**Experimental groups**			***P*-value**
	**CON**	**MGOsyn LOW**	**MGOsyn HIGH**	
**Bacteria**				
Shannon	3.69 ± 0.03^a^	3.45 ± 0.01^b^	3.23 ± 0.12^c^	< 0.0001
Simpson	0.94 ± 0.00^a^	0.94 ± 0.00^a^	0.93 ± 0.01^b^	< 0.0001
**Archaea**				
Shannon	0.48 ± 0.04^a^	0.02 ± 0.00^c^	0.26 ± 0.08^b^	< 0.0001
Simpson	0.29 ± 0.02^a^	0.01 ± 0.00^c^	0.13 ± 0.04^b^	< 0.0001

^a, b, c^Values within the same row with different superscripts are significantly different (*P* < 0.05).

**Table 3:** Relative abundance of the top 15 taxa.

**Taxa**	**Experimental groups (%)**			***P*-value**
	**CON**	**MGOsyn LOW**	**MGOsyn HIGH**	
*Rikenellaceae* RC9	16.44 ± 0.81^a^	12.50 ± 0.53^b^	5.78 ± 2.08^c^	< 0.0001
*Candidatus Saccharimonas*	2.79 ± 0.24^b^	8.06 ± 0.79^a^	9.95 ± 1.59^a^	< 0.0001
*Succiniclasticum*	0.70 ± 0.22^c^	7.09 ± 0.78^b^	12.43 ± 1.15^a^	< 0.0001
*Succinivibrio*	0.88 ± 0.22^b^	7.21 ± 1.53^a^	10.22 ± 3.56^a^	< 0.0001
*Ruminobacter*	0.40 ± 0.11^c^	12.05 ± 0.93^a^	5.28 ± 1.58^b^	< 0.0001
*Prevotella* 1	12.03 ± 0.61^a^	1.31 ± 0.16^b^	0.28 ± 0.13^c^	< 0.0001
*Prevotella* 7	0.13 ± 0.33^b^	1.38 ± 0.31^b^	12.08 ± 6.76^a^	< 0.0001
*Christensenellaceae* R-7	5.90 ± 0.19^a^	3.66 ± 0.20^b^	2.84 ± 0.53^b^	< 0.0001
Unclassified *Succinivibrionaceae*	2.83 ± 0.48^b^	5.10 ± 0.27^a^	1.47 ± 0.89^b^	< 0.0001
*Ruminococcaceae* UCG-014	2.45 ± 0.23^b^	3.74 ± 0.73^a^	2.67 ± 0.53^b^	< 0.0001
*Succinivibrionaceae* UCG-002	0.48 ± 0.17^c^	1.25 ± 0.17^b^	6.78 ± 0.48^a^	< 0.0001
*Pseudobutyrivibrio*	4.80 ± 0.17^a^	0.66 ± 0.16^b^	0.13 ± 0.05^c^	< 0.0001
Unclassified *Lachnospiraceae*	1.60 ± 0.12^ns^	1.67 ± 0.12^ns^	1.96 ± 0.43^ns^	0.0948^ns^
*Desulfovibrio*	0.05 ± 0.04^b^	2.26 ± 0.30^a^	2.83 ± 0.64^a^	< 0.0001
*Ruminococcaceae* NK4A214	1.52 ± 0.23^b^	1.98 ± 0.18^a^	1.51 ± 0.37^b^	0.0031

^a, b, c^Values within the same row with different superscripts are significantly different (*P* < 0.05).

### Impact of MGOsyn supplementation on archaeal diversity and community structure

The α-diversity indices, including the Shannon (richness and evenness) and Simpson (dominance and evenness) indices of the archaeal community, were significantly influenced by MGOsyn supplementation (*P* < 0.0001; Figure 2B, Table 2). The β-diversity analysis at the genus level showed a clear divergence between MGOsyn supplemented and control samples. Similar to the bacterial communities, the archaeal profiles of the MGOsyn supplemented groups were distinctly separated from those of the control group (*F* = 30.979, *P* < 0.001; Figure 2D). This shift was accompanied by significant alterations in the archaeal community structure particularly in the abundance of the major methanogenic group, *Methanobrevibacter* (99.7 and 93.1% for MGOsyn LOW and MGOsyn HIGH, respectively), compared with the control group (82.1%; *P* < 0.0001). The relative abundance of Unclassified *Methanomethylophilaceae* decreased in the MGOsyn supplemented groups (less than 1% for both MGOsyn LOW and MGOsyn HIGH) compared with that in the control group (17.5%; *P* < 0.0001). The MGOsyn HIGH group exhibited a higher proportion of *Methanosphaera* (6.4%), whereas this genus remained minimally represented in the control group (< 1%; *P* < 0.0001; Figure 4D).

### Correlations between VFAs, A:P ratio, CH_4_ production, and MGOsyn supplementation and bacterial genera

**Figure 5:**
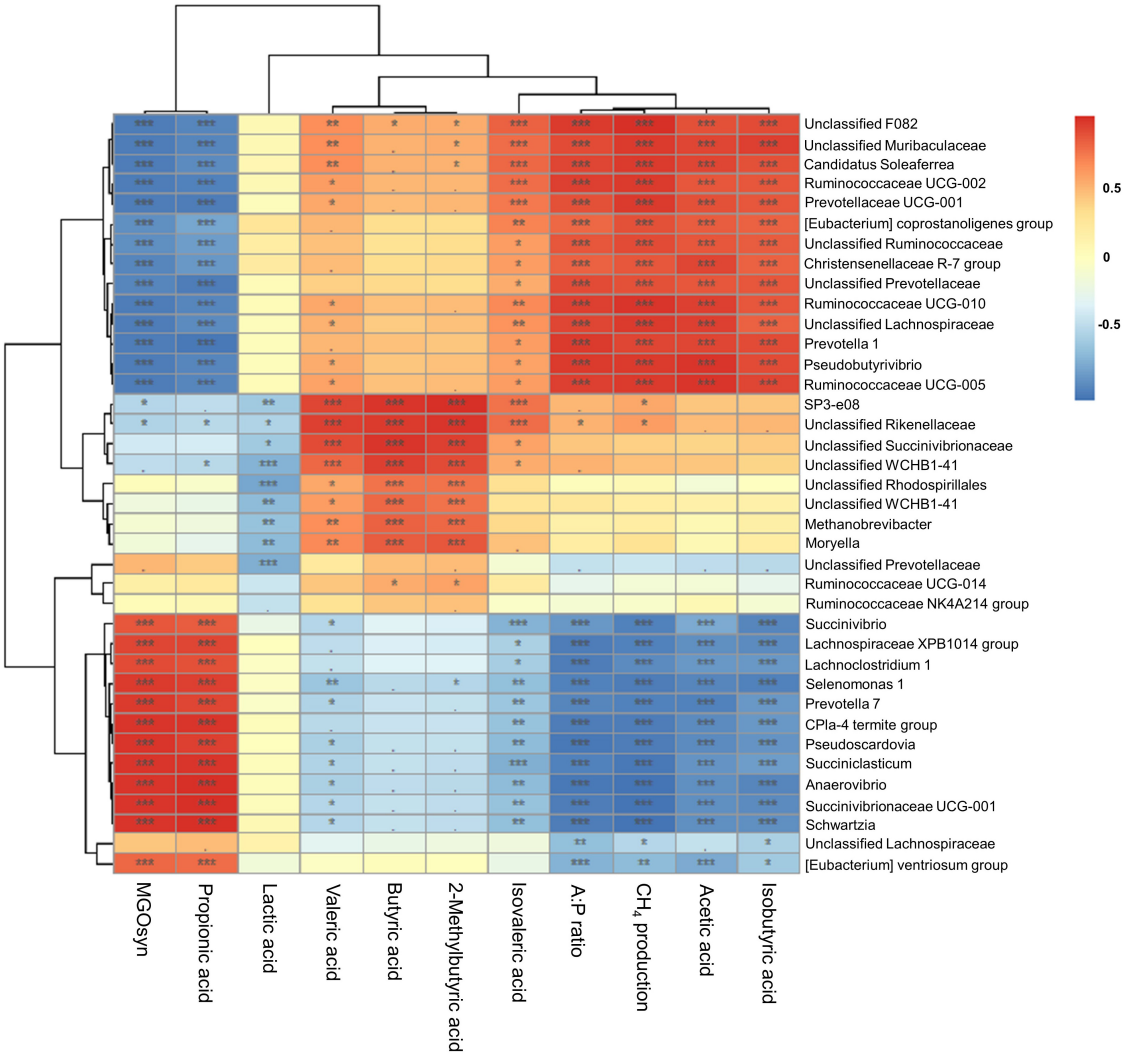
Correlations between bacterial genera and rumen fermentation parameters. Heatmap illustrating the Spearman correlation coefficients between the relative abundances of bacterial genera and key fermentation parameters: individual VFAs (acetic, propionic, butyric, isobutyric, 2-methylbutyric, valeric, and isovaleric acids), A:P ratio, CH_4_ production, and MGOsyn supplementation. Positive and negative correlations are shown in red and blue, respectively, with color intensity representing the strength of the correlation (*r*). Statistical significance: ***P < 0.001, **P < 0.01, *P < 0.05, ^•^*P* < 0.1.

**Figure 6:**
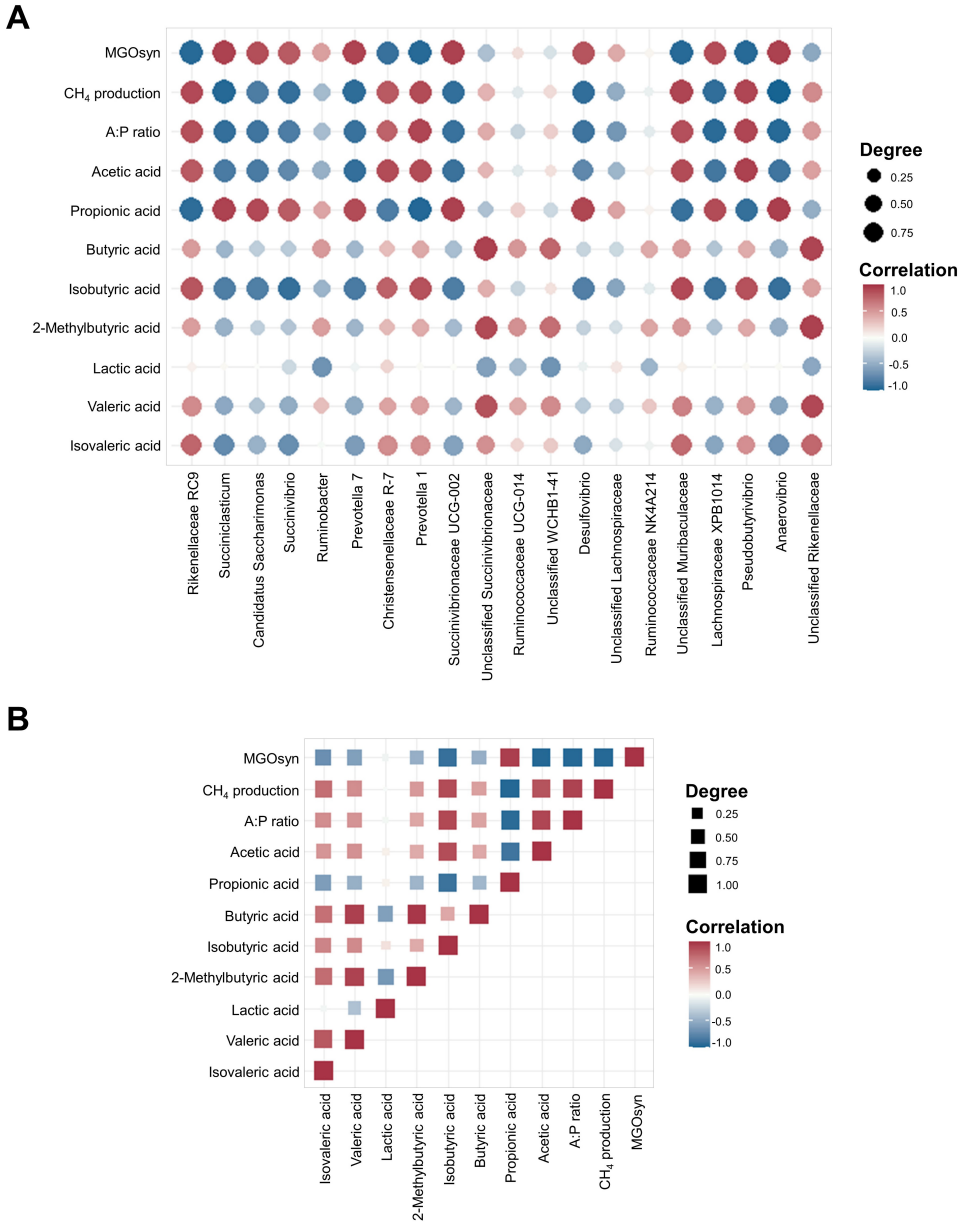
Spearman correlation coefficients between the top 20 bacterial genera and rumen fermentation parameters and intercorrelations among rumen fermentation parameters. **(A)** Correlation matrix showing the Spearman correlation coefficients between the relative abundances of the 20 most abundant bacterial genera and key rumen fermentation parameters, including individual VFAs (acetic, propionic, butyric, isobutyric, 2-methylbutyric, valeric, and isovaleric acids), A:P ratio, CH_4_ production, and MGOsyn supplementation. **(B)** Spearman correlation matrix illustrating intercorrelations among major rumen fermentation parameters, including VFAs, A:P ratio, CH_4_ production, and MGOsyn supplementation. Correlation coefficients range from −1 to 1, with the strongest positive (*r* = 1) and negative (*r* = −1) correlations indicated in red and blue, respectively. Color intensity corresponds to the magnitude of the correlation.

We conducted Spearman correlation analysis to examine the associations between the VFAs, A:P ratio, CH_4_ production, and MGOsyn supplementation and bacterial genera at the genus level. *Succiniclasticum, Candidatus Saccharimonas, Succinivibrio, Succinivibrionaceae* UCG-001, *Succinivibrionaceae* UCG-002, *Selenomonas* 1, and *Prevotella* 7 which are succinate/propionate producing bacteria, showed strong positive correlations with MGOsyn supplementation and propionic acid production, and strong negative correlations with acetic acid production, A:P ratio, and CH_4_ production. In contrast, *Rikenellaceae* RC9, *Christensenellaceae* R-7, *Prevotella* 1, and *Pseudobutyrivibrio* showed strong positive correlations with acetic acid production, A:P ratio, and CH_4_ production and strong negative correlations with MGOsyn supplementation and propionic acid production (Figures 5, 6A). Correlation coefficients between VFAs, A:P ratio, CH_4_ production, MGOsyn supplementation, and bacterial genera were calculated, and the results are summarized in Supplementary Table S2. These correlations provide an overview of the relationships among fermentation parameters, methane production, and changes in bacterial communities associated with MGOsyn supplementation.

Spearman correlation analysis was performed to examine the relationships among VFAs, the A:P ratio, CH_4_ production, and MGOsyn supplementation (Figure 6B). The intercorrelation analysis revealed a strong positive association between MGOsyn supplementation and propionic acid. In contrast, acetic, butyric, isobutyric, 2-methylbutyric, valeric, and isovaleric acids were negatively correlated with MGOsyn supplementation, whereas lactic acid showed no significant correlation (Figure 6B).

### Network analysis at the genus level of MGOsyn supplemented microbial communities

**Figure 7:**
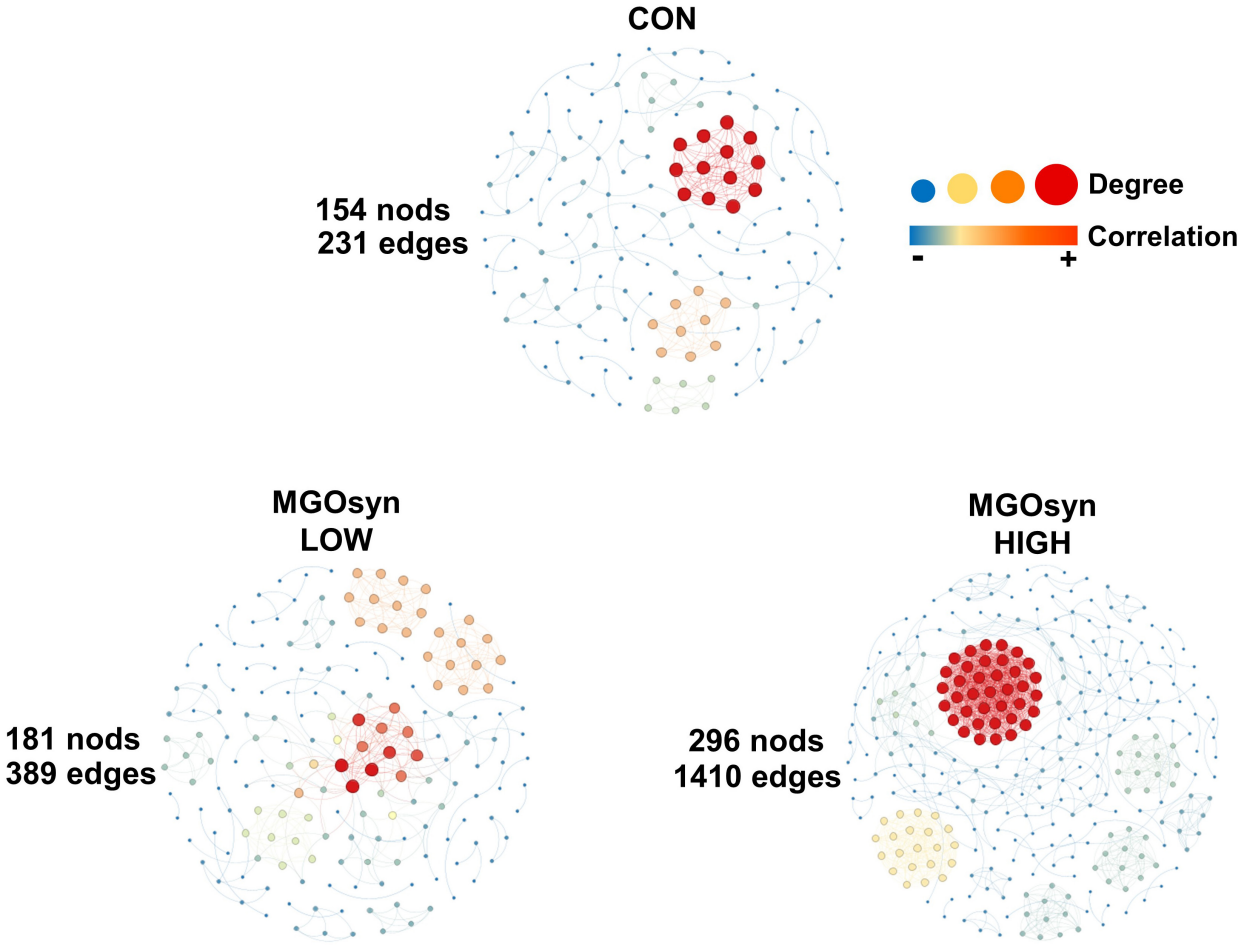
Network analysis at the genus level of MGOsyn supplemented microbial communities. Spearman rank correlations illustrating the potential interactions within the microbial communities. Only statistically significant Spearman correlations (*P* < 0.05) are included in the network visualization. Positive and negative correlations are represented by red and blue edges, respectively. Spearman correlation analyses were conducted using R software and the microbial interaction network was visualized and constructed using Gephi version 0.10.1.

We conducted network analysis based on Spearman's rank correlation at the genus level to investigate microbial communities associated with MGOsyn supplementation (Figure 7). A stronger microbial network was observed with increasing doses. The MGOsyn LOW supplemented group exhibited 181 nodes and 389 edges, whereas the MGOsyn HIGH supplemented group showed a more complex network with 296 nodes and 1,410 edges. In contrast, the control group contained 154 nodes and 231 edges (Figure 7, Supplementary Tables S3–S8).

### KEGG functional pathways of MGOsyn supplemented groups

We predicted the functional pathways in the MGOsyn supplemented groups using PICRUSt analysis. MaAsLin3 analysis of the Kyoto Encyclopedia of Genes and Genomes (KEGG) pathways revealed significant differences among the MGOsyn supplemented groups (Supplementary Figure S3). Specifically, MaAsLin3 predicted that METHANOGENESIS–PWY (methanogenesis from H_2_ and CO_2_) and PWY−6148 (tetrahydromethanopterin biosynthesis), both associated with methanogenesis related pathways, were present at significantly lower levels in the MGOsyn HIGH group (coefficients: −5.5 and −4.8, respectively). In addition, PWY−5677 (succinate fermentation to butanoate) was significantly reduced in both the MGOsyn LOW and HIGH groups (coefficients: −1.5 and −4.5, respectively; Supplementary Figure S3).

### Intercorrelations between bacterial genera

**Figure 8:**
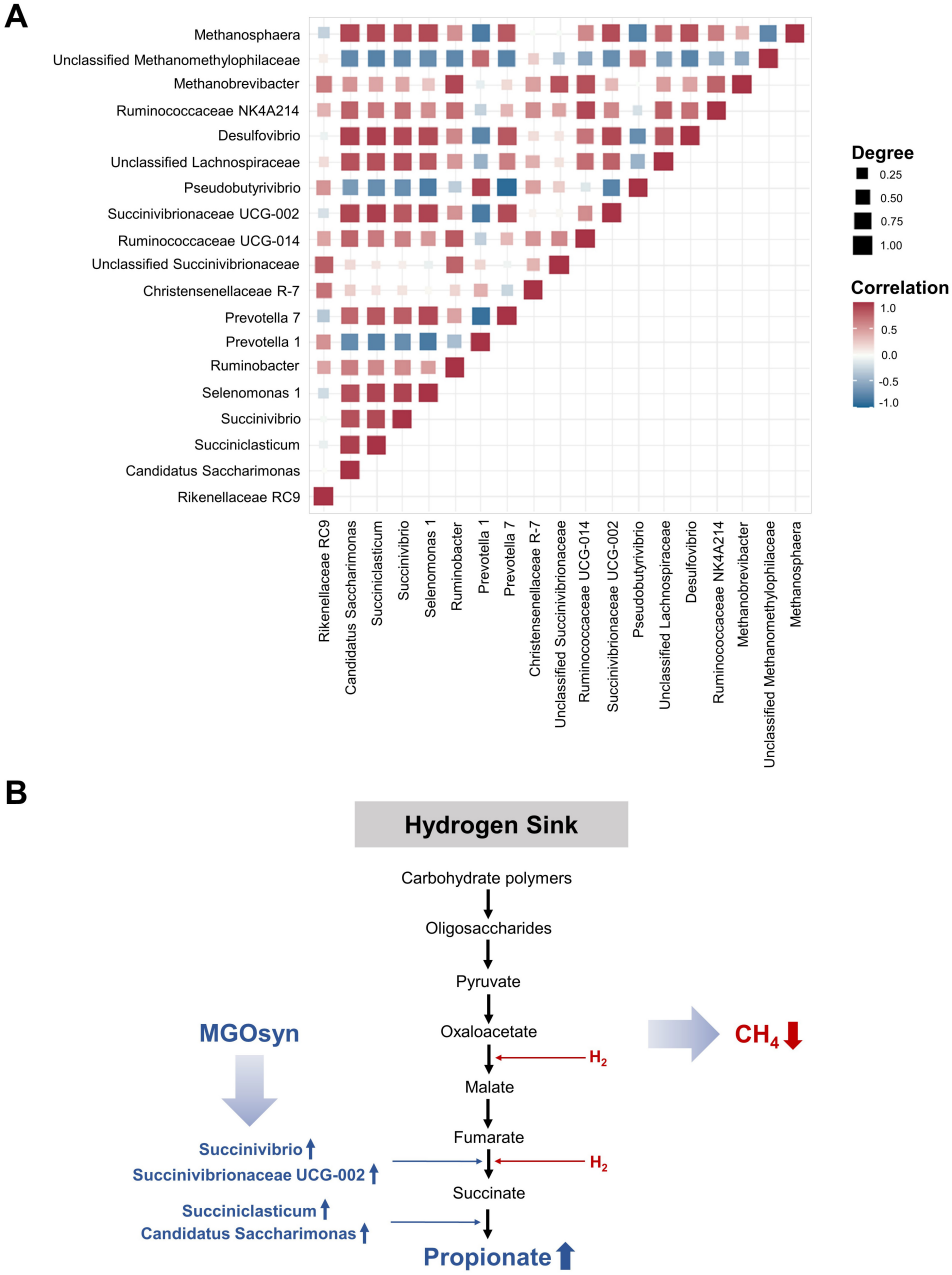
Mode of action of MGOsyn. **(A)** Spearman correlation matrix illustrating intercorrelations among 19 bacterial and archaeal genera associated with MGOsyn supplementation. Strong positive correlations (*r* = 1) are depicted in red, indicating closely cooccurring taxa, whereas strong negative correlations (*r* = −1) are indicated in blue, representing mutually exclusive or inversely related taxa. This correlation network illustrates potential microbial interactions and community dynamics influenced by MGOsyn treatment. **(B)** Schematic representation of the predicted mode of action of MGOsyn. MGOsyn is found to enhance the hydrogen sink within the rumen by promoting propionic acid production pathway, which competes for metabolic hydrogen, thereby reducing the substrate availability for methanogenic archaea. This shift results in decreased CH_4_ emissions and improved fermentation efficiency, contributing to both environmental CH_4_ mitigation and enhanced ruminant productivity.

We conducted Spearman correlation analysis to examine the intercorrelations among bacterial genera (Figure 8). Genera enriched within the MGOsyn supplemented group, including *Succiniclasticum, Candidatus Saccharimonas, Succinivibrio, Succinivibrionaceae* UCG-002, *Selenomonas* 1, *Prevotella* 7, *Desulfovibrio*, and *Methanosphaera*, exhibited strong positive intercorrelations. Similarly, genera enriched in the control group, such as *Rikenellaceae* RC9, *Prevotella* 1, *Christensenellaceae* R-7, *Pseudobutyrivibrio*, and unclassified *Methanomethylophilaceae*, also showed strong positive intercorrelations within the group (Figure 8).

## Discussion

Both *in vivo* and *in vitro* studies have demonstrated that lauric acid, garlic, and oregano compounds can inhibit methane (CH_4_) production (Kim et al., 2018; Ma et al., 2016; Panthee et al., 2017; Tseten et al., 2022). However, only a few studies have investigated the CH_4_ reducing effects of monolaurin, a compound derived from glycerol and lauric acid (Klevenhusen et al., 2009), and its mechanism of action remains largely unexplored.

Previous *in vitro* studies have demonstrated that garlic oil can reduce methane emissions by 23–43%, while oregano oil has achieved reductions ranging from 13 to 87%, depending on the concentration (Patra and Yu, 2012; Tseten et al., 2022). In the present study, we employed garlic and oregano in powdered form rather than as essential oils and observed concentration dependent reductions in methane production, ranging from 13 to 81% for garlic powder and 23–80% for oregano powder (Supplementary Figure S4). Notably, the combination of monolaurin, garlic, and oregano, formulated as MGOsyn, exhibited a synergistic effect on methane mitigation, without negatively impacting rumen fermentation characteristics (Supplementary Figure S5). These findings highlight the potential of MGOsyn as a practical feed additive for methane reduction. Nevertheless, further in depth studies are warranted to clarify the precise mechanisms underlying the action of each component and to investigate their interactions within the complex rumen microbial ecosystem.

We conducted a detailed investigation of *in vitro* rumen fermentation profiles using MGOsyn (a synergistic blend of monolaurin, garlic, and oregano) including both bacterial and archaeal community analyses. Furthermore, we extended our analysis to include correlation and intercorrelation analyses among VFAs, A:P ratio, CH_4_ production, MGOsyn supplementation, and bacterial genera. Network and functional analyses were conducted to elucidate the effects of MGOsyn supplementation. In past studies, achieving significant methane mitigation with antimethanogenic compounds typically required high doses that negatively influenced rumen fermentation parameters (Patra and Yu, 2015; Patra and Yu, 2013; Yang et al., 2007). Increased propionic acid production following MGOsyn treatment may be linked to the significant enrichment of bacterial taxa such as *Succiniclasticum, Candidatus Saccharimonas, Succinivibrio, Succinivibrionaceae* UCG-002, *Selenomonas* 1, and *Prevotella* 7 which are involved in succinate and propionate production. Many studies have demonstrated that fermentation that favors increased propionate production is strongly associated with decreased CH_4_ emissions. For example, Ungerfeld (2015) reported that CH_4_ reduction in batch cultures redirects metabolic hydrogen (H_2_) toward propionate synthesis. In a study by Kittelmann et al. (2014) observed higher propionic acid levels in cows with low CH_4_ emissions. Garlic oil supplementation in a continuous fermentation system resulted in reductions in both acetate concentration and the A:P ratio (Busquet et al., 2005) as well as with garlic powder supplementation (Wanapat et al., 2008). The simultaneous rise in propionate and decline in acetate suggests that hydrogen is being utilized for propionate synthesis rather than methane production (Wang et al., 2018). Supplementation with MGOsyn proved highly effective, reducing the proportion of methane in the generated gas by up to 61% in the *in vitro* study and altering the bacterial community composition. By analyzing the bacterial community in ruminants, the genera *Succiniclasticum, Candidatus Saccharimonas, Succinivibrio, Succinivibrionaceae* UCG-002, and *Prevotella* 7 were found to produce propionate through the randomizing (succinate) pathway by carbohydrate fermentation (Jeong et al., 2024). Alternative hydrogen consuming pathways such as propionate formation are activated competitively utilizing hydrogen that would otherwise be used for methanogenesis and thereby leading to a reduction in methane production (Pereira et al., 2022). *Selenomonas* is a known propionate producing bacterium that collaborates with lactate utilizing bacteria such as *Megasphaera elsdenii* and has been associated with low methane emission in sheep (Denman et al., 2015; Stewart et al., 2019). MGOsyn treatment resulted in a dose dependent increase in *Prevotella* 7 and a decrease in *Prevotella* 1. A previous study in sheep showed that feeding biofermented rice straw rich in *Prevotella* increased propionate production and reduced methane emissions (Kyawt et al., 2024), which supports our observations. Although that study did not identify *Prevotella* at the species level, our correlation analysis suggests that *Prevotella* 7 may contribute to propionate synthesis and methane mitigation, whereas *Prevotella* 1 may play an opposing role. These findings indicate potential functional divergence within the *Prevotella* genus and underscore the need for species level investigations to better understand their roles in rumen fermentation.

Using MaAsLin3 analysis of the KEGG pathways, we predicted that the METHANOGENESIS–PWY (methanogenesis from H_2_ and CO_2_) and PWY−6148 (tetrahydromethanopterin biosynthesis), both associated with methanogenesis related pathways, were present at significantly lower levels in the MGOsyn HIGH group. This observation was consistent with the decreased methane production measured during *in vitro* rumen fermentation in MGOsyn treated groups. Similar to its effects on the bacterial community, MGOsyn altered the archaeal community composition. Archaeal β-diversity showed significant changes with MGOsyn supplementation, mirroring those observed in the bacterial community. Archaeal sequences were predominantly assigned to the genus *Methanobrevibacter*, consistent with the results of previous studies (Deusch et al., 2017; Difford et al., 2018; Seedorf et al., 2015), which are major methane producers in the rumen (Danielsson et al., 2012; Henderson et al., 2015). MGOsyn supplementation caused dose dependent shifts in the archaeal community; *Unclassified Methanomethylophilaceae* decreased in both low and high MGOsyn treatments, whereas *Methanosphaera* increased only with high dose MGOsyn supplementation. Interestingly, *Methanosphaera* increased while *Methanomethylophilaceae* decreased in response to MGOsyn supplementation. Both taxa are methylotrophic methanogens that utilize methylated compounds such as methanol for methane production, but their contrasting patterns may indicate a shift in substrate utilization or hydrogen flow under altered fermentation conditions. Such community restructuring among methanogens may reflect potential adaptive responses to changes in available substrates or fermentation end products induced by MGOsyn. A previous study also reported that cows with high rumination time (HIGH-RT), which exhibited 26% lower enteric methane emissions, had a greater abundance of *Methanosphaera stadtmanae* (Castaneda et al., 2025), suggesting that increased methylotrophic methanogenesis associated with higher *Methanosphaera* abundance can be linked to reduced total methane output. Therefore, the observed increase in *Methanosphaera* may represent a compositional adjustment of methanogenic populations under modified ruminal fermentation dynamics. Metabolically, these cows also had higher propionate concentrations and were enriched with rapid fermenting bacteria such as *Prevotella, Sharpea, Veillonellaceae*, and *Succinivibrionaceae* (Castaneda et al., 2025). This finding supports our correlation analysis, in which *Methanosphaera* showed strong positive correlations with propionate producing bacteria including *Succiniclasticum, Candidatus Saccharimonas, Succinivibrio*, and *Succinivibrionaceae* UCG-002. Further studies are needed to elucidate the mechanisms underlying the interactions among archaea, bacteria, and metabolites in the rumen environment.

Functional predictions derived from microbial community analyses provide only potential insights into metabolic activities, as they are inferred from taxonomic composition and relative abundances rather than from direct measurements of functional processes. Consequently, such predictions serve as exploratory indications rather than definitive evidence of microbial function. In this study, we addressed these limitations by comparing predicted functions with actual measurements of VFAs metabolism, allowing a more accurate assessment of fermentation processes and a deeper understanding of MGOsyn's effects on rumen microbial activity. Nevertheless, we acknowledge the inherent limitations of *in vitro* systems, including the absence of host interactions and the potential for experimental artifacts. Based on these exploratory *in vitro* results, further *in vivo* studies are warranted to validate the findings and to comprehensively evaluate the efficacy of MGOsyn under physiological conditions.

This study serves as a valuable example of predicting the methane reducing effects of a novel natural additive through an *in vitro* rumen fermentation screening process prior to conducting *in vivo* trials in actual cattle. By analyzing VFAs and the microbiome of the *in vitro* fermented rumen samples, the study not only interpreted the significance of each result individually but also explored their interrelationships, allowing prediction and inference of potential methane reduction pathways in ruminants. Taken together, these findings provide foundational data to support future *in vivo* research and indicate that MGOsyn, as a natural feed additive, holds strong potential for practical application in economically feasible methane reduction approaches.

## Conclusion

MGOsyn supplementation reduced the proportion of CH_4_ in a dose dependent manner. It shifted rumen fermentation toward increased propionate production, decreased acetate production, and resulted in a lower acetate to propionate (A:P) ratio. Additionally, MGOsyn effectively increased the abundance of hydrogen consuming or propionate producing bacterial groups, including *Succiniclasticum, Candidatus Saccharimonas, Succinivibrio, Succinivibrionaceae* UCG-002, and *Prevotella* 7. The archaeal community composition was also altered, with a reduction in *unclassified Methanomethylophilaceae* observed in both low and high MGOsyn treatments, while *Methanosphaera* was increased only in the MGOsyn HIGH group. To further investigate the mechanisms underlying MGOsyn effects, we performed correlation and intercorrelation analyses between fermentation parameters and microbial taxa and conducted functional predictions of relevant metabolic pathways based on the microbial profiles. These integrative analyses provided valuable insights into how MGOsyn modulates microbial metabolic activity, particularly those involved in hydrogen utilization and propionate production, ultimately contributing to methane mitigation. Taken together, these findings suggest that MGOsyn, as a novel natural feed additive, has strong potential to reduce CH_4_ emissions from ruminants. Moreover, the enhancement of propionate synthesis via the hydrogen sink pathway may contribute to improved fermentation efficiency and livestock productivity.

## Data Availability

The data presented in this study are publicly available. This data can be found at: https://www.ncbi.nlm.nih.gov/sra, accession PRJNA1313786.
