# Supplementary material for: Effects of the monolaurin based feed additive MGOsyn on methane production, rumen fermentation, and microbial communities using rumen fluid from Hanwoo steers in an in vitro study
Source: Front Microbiol. 2025 Nov 10;16:1699688. doi: 10.3389/fmicb.2025.1699688 (PMC12643466; doi:10.3389/fmicb.2025.1699688)
Supplement: Supplementary file 1 [file Supplementary_file_1.docx]

**Supplementary information**

Effects of the Monolaurin Based Feed Additive MGOsyn on Methane Production, Rumen Fermentation, and Microbial Communities Using Rumen Fluid from Hanwoo Steers in an In Vitro Study

Mi Ae Park^1^, Seoyun Son^1^, Da Jung Lim^1^, Hee Seop Yu^2^, Yong Hee Yoon^2^, Seon-Ho Kim^3^, Sang-Suk Lee^3^, Dae-Hyuk Kim^1,4^, and Yangseon Kim^1*^

^1^Department of Research and Development, Center for Industrialization of Agricultural and Livestock Microorganisms, Jeongeup-si, Jeollabuk-do, 56212, Republic of Korea

^2^Jungnongbio, Jeongeup-si, Jeollabuk-do, 56212, Republic of Korea

^3^Department of Animal Science and Technology, Sunchon National University, Suncheon-si, Jeollanam-do, 57922, Republic of Korea

^4^Department of Bioactive Material Sciences, Jeonbuk National University, Jeonju-si, Jeollabuk-do, 54896, Republic of Korea


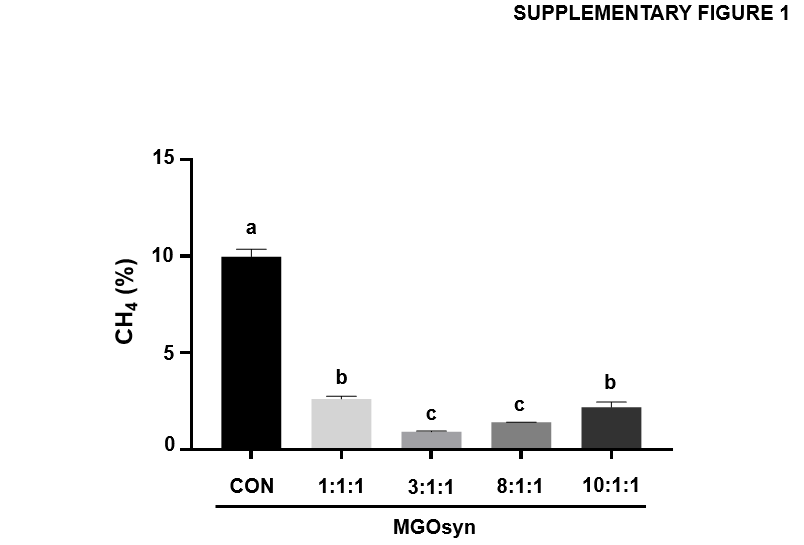


**SUPPLEMENTARY FIGURE 1. Methane production at different MGOsyn combination ratios in an *in vitro* rumen fermentation.** MGOsyn was tested at a 0.2% inclusion level with four component ratios of monolaurin:garlic:oregano (1:1:1, 3:1:1, 8:1:1, and 10:1:1) to evaluate the optimal combination for methane mitigation. Different letters above the bars indicate significant differences among treatments (*p* < 0.05).


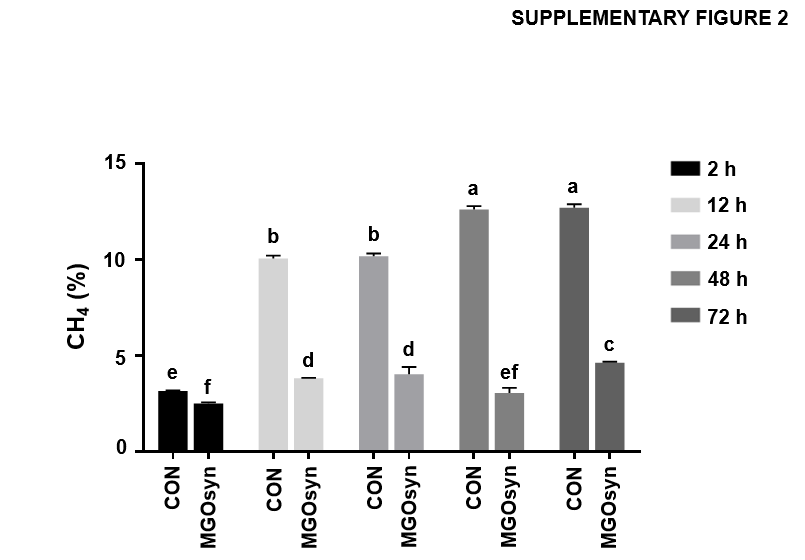


**SUPPLEMENTARY FIGURE 2.** **Effects of MGOsyn on methane production over time.** The percentage of methane (CH₄) in the total gas produced after 2, 12, 24, 48, or 72 h of *in vitro* rumen fermentation with MGOsyn supplementation. Bars with different superscript letters indicate statistically significant differences among treatment groups (*P* < 0.05).

**
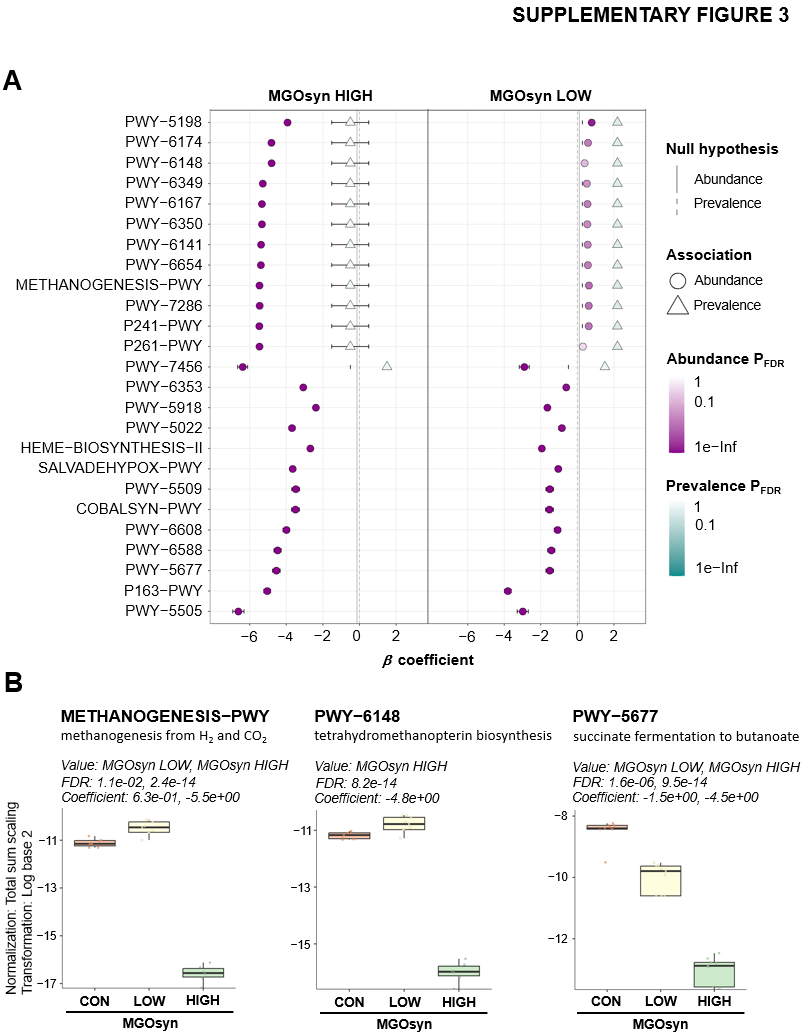
**

**SUPPLEMENTARY FIGURE 3. Predicted functional pathways affected by MGOsyn supplementation.** (A) Results of MaAsLin3 analysis based on PICRUSt predicted functional pathways. The plot shows associations of metabolic pathways with MGOsyn supplemented groups (LOW and HIGH) compared with the control (CON). (B) Relative abundances of key functional pathways significantly affected by MGOsyn supplementation, including METHANOGENESIS–PWY, PWY–6148, PWY–5677. Boxplots show pathway abundances across the CON, MGOsyn LOW, and MGOsyn HIGH groups.


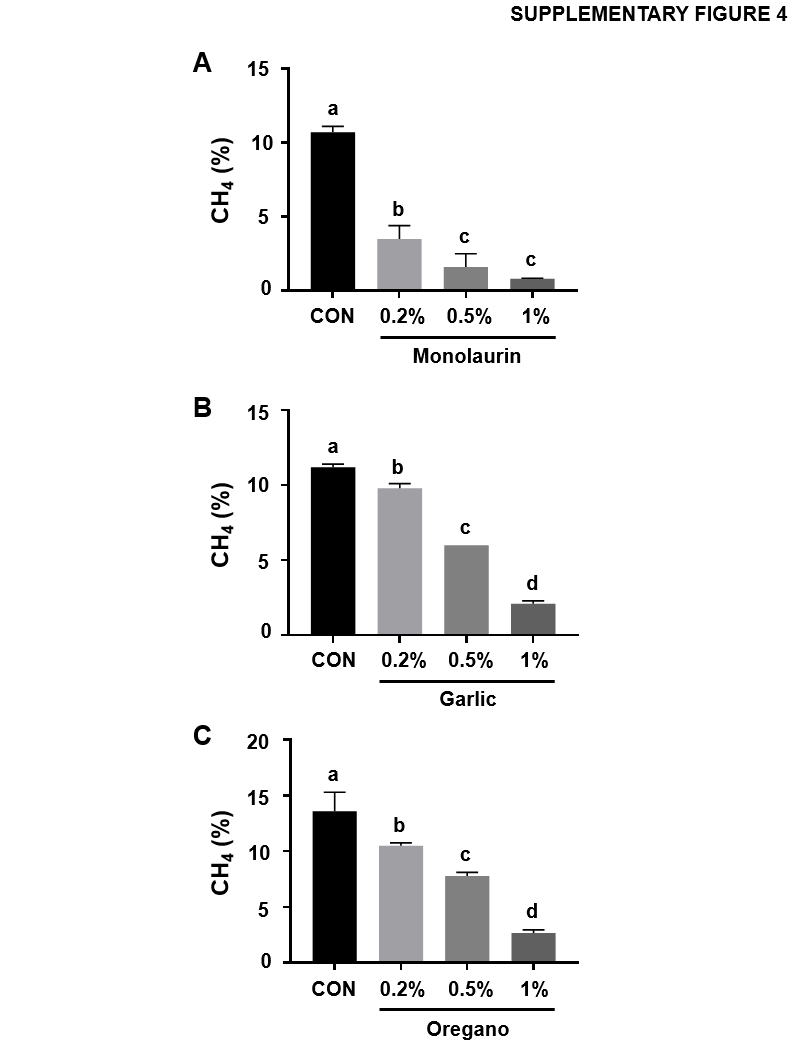


**SUPPLEMENTARY FIGURE 4. Effects of monolaurin, garlic, and oregano on methane production.** Percentage of methane (CH₄) in the total gas produced after 24 h of *in vitro* rumen fermentation with 0.2%, 0.5%, or 1% supplementation of **(A)** monolaurin, **(B)** garlic, and **(C)** oregano. Bars with different superscript letters indicate statistically significant differences among treatment groups (*P* < 0.05).


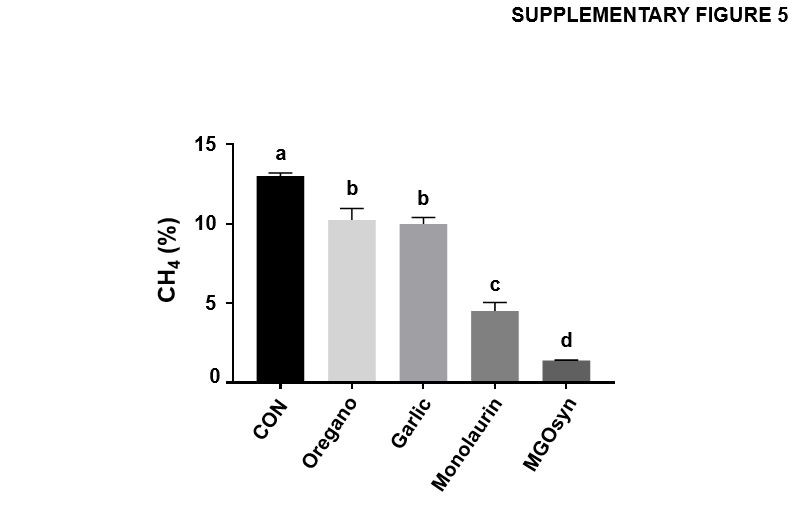


**SUPPLEMENTARY FIGURE 5.** **The combination of monolaurin, garlic, and oregano exhibited a synergistic effect in reducing CH₄ emissions.** The percentage of methane (CH₄) in the total gas produced was measured after 24 h of *in vitro* rumen fermentation with 0.2% supplementation of monolaurin, garlic, oregano, or MGOsyn. Bars with different superscript letters indicate statistically significant differences among treatment groups (*P* < 0.05).

**SUPPLEMENTARY METHOD**

**Experimental Design and *in vitro* Rumen Fermentation**

The experimental groups were prepared based on supplementation with monolaurin, garlic, oregano, or MGOsyn at levels of 0% (CON), 0.2%, 0.5%, or 1% of the total fermentation volume. To further evaluate the optimal combination ratio, MGOsyn was tested at a 0.2% inclusion level with four different component ratios of monolaurin:garlic:oregano (1:1:1, 3:1:1, 8:1:1, and 10:1:1). Each bottle contained 1 g of ground concentrate, passed through a 1 mm sieve, as the substrate, which was placed in a 250 mL ANKOM fermentation bottle (ANKOM Technology, USA). A total of 100 mL of rumen fluid and McDougall’s buffer (1:2 ratio) was added to each bottle under continuous CO₂ flushing. The bottles were then incubated at 39°C for 2, 12, 24, 48, or 72 h. At the end of incubation, methane gas concentrations were quantitatively determined using a gas chromatography (GC) system (Nexis GC-2030, Shimadzu, Japan).
